# The marks of gunshot wounds to the face

**DOI:** 10.1016/j.bjorl.2019.07.008

**Published:** 2019-09-05

**Authors:** Adriane Batista Pires Maia, Simone Gonçalves Assis, Fernanda Mendes Lages Ribeiro, Liana Wernersbach Pinto

**Affiliations:** a Colégio Brasileiro de Cirurgia e Traumatologia Bucomaxilofacial, São Paulo, SP, Brazil; b Escola Nacional de Saúde Pública da Fundação Oswaldo Cruz (ENSP/FIOCRUZ), Departamento Latino-Americano de Estudos de Violência Sérgio Carelli, Rio de Janeiro, RJ, Brazil; c Hospital Central da Polícia Militar do Estado do Rio de Janeiro, Rio de Janeiro, RJ, Brazil

**Keywords:** Firearm injury, Facial trauma, Oral and maxillofacial surgery, Maxillofacial fracture

## Abstract

**Introduction:**

This article deals with the occurrence of health problems due to gunshot wounds to the face among military police officers, in the metropolitan region of Rio de Janeiro, who were submitted to surgery at the Oral and Maxillofacial Surgery and Traumatology Clinic of Hospital Central da Polícia Militar.

**Objective:**

To identify the profile of patients submitted to surgery as a result of gunshot wounds, the anatomical distribution of maxillofacial fractures, the identified sequelae and complications, the health specialties involved in the rehabilitation of these patients, and to discuss the social, emotional and work performance-related effects of work among these subjects.

**Methods:**

A retrospective epidemiological study was carried out based on secondary data from military police officers who were submitted to surgery at Hospital Central da Polícia Militar due to gunshot wounds from June 2003 to December 2017.

**Results:**

During the study period, 778 surgeries were performed in the operating room by the Oral and Maxillofacial Surgery and Traumatology service at Hospital Central da Polícia Militar, 186 of which were due to gunshot wounds (23.9%). All patients were males and the mean age 34.7 years. Bone segment loss was the most common sequela. Facial esthetic impairment and reports of insomnia were the most often identified late consequences of impact on health and social life. Regarding the occupational impacts of the sustained injury, the mean time away from work due to medical leave for the treatment of maxillofacial injuries was 11.7 months.

**Conclusion:**

The treatment of gunshot wounds patients with facial injuries requires multiple surgical interventions and their rehabilitation requires the involvement of different health specialties. Further studies are needed to qualitatively analyze the impact of this type of facial trauma on the patients' lives and their social consequences.

## Introduction

The article assesses the occurrence of health problems caused by non-fatal gunshot wounds (GSW) to the face that affect military police (MP) personnel in the state of Rio de Janeiro, Brazil, during their work activities and outside them. It points out the scarcity of studies and viewpoints that analyze the social, emotional and work-related effects. In addition to the physical sequelae, body esthetics in the makeup of today’s individuals are especially important, since the face is a very consequential element in the development of individualism.^1^, ^2^

Brazil is a country historically marked by violence.^3^ Between 1980 and 2014, 967,851 people died from gunshot wounds, which places Brazil among the 10 countries with the highest rates of firearm homicides worldwide.^4^ This type of social violence also affects the military police in the country.^5^ Rio de Janeiro was the state with the highest absolute number of military police officers killed^6^; however, the numbers of firearm injury cases in this class of workers are not known, which hinders the understanding of the real magnitude of this problem. According to Maia’s^7^ systematic review article, the firearm is the main mechanism of injury at work among police officers, more frequent among male security professionals aged approximately 31 years. Because of the potential of this trauma to cause morbidity and mortality, along with the increase in the prevalence of this type of injury due to the use of weapons with high potential for destruction that affects the civilian population and public security professionals, this problem is relevant for areas including Medicine, Dentistry and Public Health.

GSW to the face result in blunt puncture wounds and avulsion lesions of the maxillofacial segments, which usually result in the most devastating type of facial trauma, constituting a challenge for the surgeon.^8^ GSW, in addition to the entrance and exit orifices, are characterized by cavitation formation. Cavitation is the lateral leak of tissues that form during the passage of the projectile, initially filled with water vapor, called temporary cavities.^9^ As the lesion retracts, it results in what is termed a permanent cavity. The dimensions of the permanent cavity are determined by the tissue density affected by the projectile, its shape and velocity. Facial lesions produce temporary cavities that result in emphysema and significant edema, representing a risk of airway impairment minutes after the trauma, in addition to increasing the risk of infections.^10^

Due to the complexity of this type of trauma, treatment involves multidisciplinary teams, multiple reconstructive surgeries, extensive recovery periods and physical limitations that invariably also affect the social and emotional aspects of the patient's life.^11^, ^12^, ^13^, ^14^, ^15^ It is hypothesized that the esthetic consequences resulting from trauma to the face are important elements that have an impact on mental health and may aggravate the psychological changes. Nevertheless, few studies have sought to investigate and describe the location of these injuries and the impact on the police officers’ health, both for professional performance and their social life. This study, therefore, is original in investigating a gap in scientific research, by addressing, in addition to the physical effects, the consequences for work and their social relations.

## Methods

This descriptive study was carried out based on the survey of secondary data regarding military police officers submitted to surgery at Hospital Central da Polícia Militar from June 2003 to December 2017. All active military police officers operated for GSW under general anesthesia in the Oral-Maxillofacial Surgery and Traumatology Service (CTBMF) at Hospital Central da Polícia Militar, regardless of the injury severity, were included in the analysis. The study excluded civilian patients, retired military police officers and those working who received a GSW to the face but did not require surgery under general anesthesia or who underwent surgery in other hospitals of the state. The study shows the frequency and distribution of firearm wounds in the population of police officers in the State of Rio de Janeiro who did not immediately die as a result of the injury. Thus, 186 surgeries performed on 77 patients met the inclusion criteria and were included in the study. The research was submitted to and approved by the Research Ethics Committee of Fundação Osvaldo Cruz in 2018 under Opinion number CAAE 81841317.6.0000.5240.

Patient epidemiological information includes age, gender, ethnicity/skin color, and date of trauma. As for the work-related variables, the following were analyzed: military rank, unit where the MP was stationed, if the injury occurred on duty or on time off.

The maxillofacial injuries were classified according to their location, whether intraoral or extraoral (frontal bone to the mandible), indicated whether the injuries produced fractures of the facial bones and their location, distributed by region: mandibular, maxillary, zygomatic-orbital, nasal and frontal. The number of surgeries performed after the trauma, the most common complications and health specialties involved in rehabilitation were also indicated.

Regarding the consequences of the injury, we analyzed: the complications and sequelae resulting from the trauma, the time of medical leave to treat GSW in the face, whether the patient could return to work as Category A — Fit for Work (without restrictions), Category B — Fit for Work (with physical restrictions) or Category C — Fit for Work (with firearm use restriction), or whether the MP officer was considered temporarily or permanently unfit for work and must be retired or sent to the reserve.

Data collection was performed using a standardized form based on the variables obtained from the service records, entered into a database (Epidata 3.0 Program), according to the established criteria. For data processing and analysis, the statistical package SPSS version 19.0 was used, describing the frequency and percentage.

## Results

During the study period, 778 surgeries were performed in the operating room by the CTBMF service at Hospital Central da Polícia Militar. Of this total, 186 surgeries were performed due to gunshot wounds, representing 23.9% of all procedures under general anesthesia in the service. The 186 surgeries were performed on 77 active military police officers; a mean of 2.4 surgeries per patient for the treatment of injuries and fractures caused by GSW to the face.

All patients with GSW to the face in this analysis were males, aged 24–48 years, with a mean age of 34.7 years. It was not possible to identify ethnicity/skin color in 54.5% of the records. Of the 35 assessed patients, 51.4% were identified as White, followed by Black (28.5%) and Brown (20%).

The second quarter of the year was the period with the highest occurrence of this type of surgery. The year 2017 was the one with the highest frequency (n = 10) of surgeries in patients with a GSW to the face. The professional profile of the police officer consisted predominantly of the lower ranks (97.3%); especially soldiers (41.6%); 69.9% of the cases occurred during the work period; 88.2% of the military police officers were, at the moment, exercising the main activity.

Among the circumstances involved in the accidents while off duty, mugging or attempted mugging was the most often reported occurrence (72.7%); followed by suicide attempt (9.1%).

Regarding the oral and maxillofacial injuries analyzed, 97.4% of the patients had extraoral injuries and 85.7% had intraoral injuries; 64.9% of the patients suffered tooth loss and 80.5% suffered facial fractures. As for the extension of the lesions, 62 patients had 109 fractured facial regions, with greater involvement of the mandible region, as reported in the literature,^17^, ^18^, ^19^, ^20^ followed by the maxilla and zygomatic-orbital complex regions (Table 1).

**Table 1:** Distribution of maxillofacial fractures due to gunshot wounds (June 2003 to December 2017, n = 109).

Oral and Maxillofacial Region	% of fractures per region (n = 109)	% of patients with impaired region (n = 62)
Mandible	33.0	58.1
Maxilla	26.6	46.8
Zygomatic-orbital	24.8	43.5
Nasal	9.2	16.1
Frontal	6.4	11.3

As a result of the GSW trauma, 18.2% of the affected MP officers had to be submitted to tracheotomy. Some type of complication was reported during hospitalization in 13.8% of the patients, with infection being the most frequent among them.

The medical records show 275 occurrences of sequelae and complications resulting from the trauma among the 77 patients in the sample (average of 3.6 occurrences per patient), as shown in Table 2. Bone segment loss was the most frequent sequela, present in 53 cases, followed by: paresthesia, infection, facial nerve palsy, masticatory function limitations, malocclusion, recurrent sinusitis, visual alterations (14), amaurosis (in 8 cases), temporomandibular disorders, orosinusal or oronasal communications, speech limitations, glandular alterations, anosmia, lacrimal apparatus alterations, rigid internal fixation failure and graft loss. The late consequences of the impact on health and social life were: esthetic facial impairment in 57 patients, reports of insomnia (30), apparent scars (63), complaints of chronic pain (32), psychological and/or psychiatric disorders (5), loss of balance and constant tinnitus in the ear (2).

**Table 2:** Distribution of sequelae and complications caused by trauma suffered by military police officers in the state of Rio de Janeiro (June 2003 to December 2017, n = 77).

Complications and sequelae	n	% according to occurrence (n = 275)	% per patient (n = 77)
Loss of bone segments	53	19.4	68.8
Paresthesia	50	18.2	64.9
Infection	29	10.5	37.7
Paralysis	26	9.5	33.8
Masticatory function limitations	24	8.7	31.2
Malocclusion	19	6.9	24.7
Recurrent sinusitis	15	5.5	19.5
Visual alterations	14	5.1	18.2
Oro-sinonasal communication	10	3.6	13.0
Temporomandibular dysfunction	10	3.6	13.0
Speech limitation	8	2.9	10.4
Glandular alterations	6	2.2	7.8
Anosmia	4	1.5	5.2
Changes in the lacrimal apparatus	3	1.1	3.9
Fixation failure	2	0.7	2.6
Loss of graft	2	0.7	2.6
Total	275	100.0	–

Such complications are expressed as functional and esthetic limitations in the patient's life, causing reduction in mastication capacity, with acute weight loss, reduction in sense of taste, partial or total loss of visual acuity, speech difficulty, among other daily life restrictions. We also know that these complications can produce esthetic changes in the face with social and emotional consequences for the patient.

Among the 77 operated patients, only 14 were not referred to another health specialty. On average, 1.8 health specialties were involved in the treatment of each patient with this type of facial wound; 63 patients received 136 referrals to 14 different specialties, according to Table 3. Regarding Dentistry, 41.6% of the patients needed this specialty, distributed into: endodontics (7), dentistic (7), prosthesis (6), implant dentistry (5) and temporomandibular dysfunction clinic (1).

**Table 3:** Distribution of specialties involved in the treatment of patients with GSW to the face (June 2003 to December 2017, n = 136).

Specialty	n
Physiotherapy	30
Psychology	26
Ophthalmology	18
Psychiatry	10
General surgery	6
Neurosurgery	5
Otorhinolaryngology	5
Cardiology	4
Plastic surgery	2
Neurology	2
Thoracic surgery	1
Hyperbaric Medicine	1
Internal Medicine	1
Dentistry	25

Among the patients who received the largest number of referrals, between 4 and 6 specialties, it was observed that all needed psychology and/or psychiatry consultations during the postoperative period.

Regarding the occupational consequences of the injury suffered, the time of medical leave for treatment was calculated based on the analysis of 47 patients whose respective records contained the necessary dated information described by the surgeon. Time away from work ranged from 1 to 60 months, with an average of 11.7 months. The mode was 6 months on medical leave. The medical records of 58 patients showed their categories regarding the return to work after the trauma: 28 received the Fit-for-work Category A classification and 9 received the Category B; another 20 were retired and one was sent to the reserve. However, it is not possible to affirm that the GSW to the maxillofacial region was the only determining factor for this condition, since the patients may have suffered other injuries during the same event or have been retired due to psychological disability.

The analysis of the medical records showed that 13 patients who underwent oral and maxillofacial surgery for gunshot wounds also were evaluated by another health specialty, including ophthalmology (9), psychiatry (2), neurology (1) and orthopedics. (1).

Surgical treatment ranged from simple irrigation and immediate and sometimes late debridement of wounds, surgeries for early reduction and stabilization of bone stumps through the use of rigid internal fixation devices, with emphasis on symmetry and function recovery, and reconstruction surgery with bone grafts.

## Discussion

The professional profile of the patient with GSW to the face found was a military police of the lower ranks, especially soldiers, with a mean age of 34.7 years; hit while on duty. Differences were observed regarding the mean age, which is older among the military when compared to civilian patients (approximately 25 years).^18^, ^21^, ^22^, ^23^, ^24^ All operated patients belonging to the Rio de Janeiro military police were males, a fact that could be expected, given the small number of female military police officers in the corporation. On the other hand, the male gender is also the most frequent to be affected by GSW in the general population, as several studies have pointed out.^4^, ^7^

Firearm-related trauma results in severe avulsion injuries,^14^, ^15^, ^20^, ^25^^,^^26^ especially in the mandible region,^17^, ^19^, ^27^ which requires several surgeries and the involvement of a multidisciplinary team and has severe consequences for the lives and work activities of those affected. Mandible fractures were the most common, followed by those in the maxilla and zygomatic-orbital regions. On average, each patient had 1.75 regions with maxillofacial fractures.

These were followed by loss of bone segments in 80.6% of cases and dysesthesias in 99%, which may be explained by the high avulsion and destructive potential of the firearm projectile. The military police officers in Rio de Janeiro do not wear personal protective equipment for the maxillofacial region, such as ballistic helmets or goggles. In fact, this type of equipment is not common among police officers worldwide, as is most commonly found among military personnel in a war situation.^28^, ^29^ It is known that such equipment can reduce damage and even prevent many maxillofacial injuries.^30^, ^31^

Regarding the late effects on the health and social life in the analyzed patients, reports of facial esthetic impairment were most commonly documented in the medical records, despite all the surgical efforts to reconstruct the damage caused by the GSW. Moreover, complaints of chronic pain and psychological disorders were frequently noted among these patients, as has also been identified in other studies analyzing the health of military police officers in Rio de Janeiro.^32^, ^33^, ^34^, ^35^, ^36^ The social impact of this type of health problem on the police officer’s face starts as soon as their family members meet their loved one in the hospital bed. The GSW produce facial edema and deformities that dramatically change the patient’s appearance.

It is not uncommon for the children of these military police officers not to recognize their parents and refuse to hug them, which creates an extremely sad and difficult situation to overcome. The path of recovery is marked by chronic pain that affects the mood and hinders emotional stability; and the wound scars indicate every day the reality and finality of the high risk of victimization of military police officers in the state of Rio de Janeiro.

Undoubtedly such morbidities to the face have consequences for the return to work; however, there is a gap in this field of study regarding such issues. Military police officers affected by GSW to the face, even after several facial reconstruction procedures, usually carry physical deformities that somehow stigmatize them as those who have been hit and marked by violence, which can unfold into changes in their social behavior. The fear of being victimized is no longer a possibility, but a reality that haunts the daily work of both the affected police officer and other coworkers, who, upon seeing the scars on the faces of their colleagues will identify the real risk resulting from the profession. This health damage is not only due to the increasing destructive power that firearms have acquired, producing difficult-to-treat sequelae, but also due to their potential to interfere with the police officer's social life, impairing their quality of life.^37^, ^38^, ^39^ The MP who is kept away from their work activities due to health reasons meets the criteria described by Ordinance number 0346; of May 12, 2010,^40^ which instructs and regulates health inspections and health inspection boards under the PMERJ.

After the medical inspection, a police officer in this situation may be considered as Category A — Fit for work — when fully capable of performing all military police services; Category B — Fit for Work, when the individual has the capacity to develop police service or activities inherent to the position or function of support activity, with restrictions for some activities clearly defined by the Health Inspection Form and may carry a firearm; Category C — Fit for work, when they manifest aptitude to develop activities inherent to the position or function of a police officer, as support activity, with restrictions to carrying a firearm. This MP may also be considered unable to carry out any Military Police activity, being considered unfit to work, and this condition may be temporary or definitive. Among patients submitted to surgery as a result of GSW, when it was possible to identify which health category was attributed to them, it was possible to verify that a substantial number of individuals can return to work, fully exercising the main activity (Category A — 48.3%); However, the percentage of retired police officers due to permanent incapacity to perform any function in the PMERJ institution was high (34.5%).

Regarding the emotional consequences for the MP victim of GSW to the face, we empirically identified a certain pattern of behavior among those affected. After the initial trauma, as soon as it was possible to establish a dialogue, the police officer is euphoric, talking, insisting in explaining in detail how it all happened, what type of position he assumed, what the scenario was at the time of its occurrence. There is a certain “joy” in having survived. Over the weeks, as this police officer becomes aware of the acquired limitations, the long trajectory to recovery that must be faced and all the changes in the work, socioeconomic and family life that the accident causes, he begins a process of introspection, loneliness, fear and depression.

Although most facial reconstructions are satisfactory from an aesthetic-functional point of view, the patient does not always overcome the trauma suffered and may express suffering and/or insecurity upon returning to work. It seems that, after they suffer such violence, something beyond the physical injury sets in and the consequences and social and professional impact of these injuries can be so profound that, despite the success of the surgical treatment, these police officers seem to be irrevocably scarred.^34^, ^35^, ^36^ Several elements seem to be involved in this. Here, we specifically focus on the significance of the impact of facial injuries, given the importance of this body part to individuals.

As of the fifteenth century, individual portraiture became one of the greatest inspirations in painting, and portraits, especially of the face, gained increasing importance over the centuries. Le Breton (2002),^2^ to explain the foundations of the modern individual formation, used the body as the starting point and the face as an element indicating the development of individualism, in which the meaning of the face changes and the body axiology alters in this modern transition. The body, including the face, gains psychological significance, just as one can read the facial expressions, which have meanings that allude directly to the subject’s personality. The physical appearance of the individual expresses his character and, therefore, must be controlled as a form of protection.^41^ The face is the most individualized, most particular part of the body, and has an important representation in the transition from the great social body to an individual body. For Elias (1994, p. 155),^1^ the face is one of the most important representations of who we are; it has perhaps a more central role in the construction of self-identity and sociability. It is this part of the body that we do not cover, and which identifies and differentiates us. The face holds our history and marks; it depicts our age, some tastes and ethnic ancestry. We can escape the mirror and the gazing directed at our bodies, but the same is practically impossible when it comes to the face. Through it, we can be identified through several official records such as the ID, the work class IDs, the club IDs, among others.

Within this perspective that considers the representativeness of the face as the “epiphany of the subject” (Le Breton, 2002, p. 41)^2^ and considering a growing process of individualization that has reached a peak with the taking of “selfies” and exposure on social media, we can surmise that individuals affected by GSW to the face potentially have an even greater impact on their lives.

The marks on one’s face tell stories and the marks we see on the faces of many military police officers in Rio de Janeiro bring out a violent context that also depicts an environment of revenge that has sentenced many to a new life, sometimes affected by restrictions in the body and at work and, almost always, undoubtedly manifested by explicit signs of violence. These subjects are required to redefine their own identity relationship with their faces, which demands a subjective work about themselves. The great relevance of referral to physiotherapy and psychological therapy indicates how rehabilitation should be seen, not only by the body health but, above all, also by mental health. Due to the lack of studies that correlate such phenomena, it is not yet possible to more fully comprehend the consequences of gunshot wounds on the lives of police and military personnel. However, by identifying the epidemiological profile of military police officers from Rio de Janeiro affected by GSW to the face, the pathways of rehabilitation treatments and the consequences for professional practice will take a step toward the understanding of the effects of this type of injury on these individuals’ working relationships.

## Conclusion

The profile of military police officers submitted to surgery following GSW to the face showed they were males, with a mean age of 34.7 years and of the lower military ranks, especially soldiers. Injuries and fractures in the mandibular region were the most common and bone segment loss was the main sequel. Patients with GSW to the face underwent two more surgeries on average and their treatment required the participation of multiple medical specialties. Facial esthetic impairment and reports of insomnia were the most frequently identified late effects on health and social life.

As a preventive measure against this type of injury, the use of personal protective equipment for the face should be considered. However, structural changes related to public safety planning need to be considered if, in fact, one intends to reduce morbidity from firearm injuries in the state of Rio de Janeiro, since no records were found in the international literature similar to the number of police officers with GSW to the face as in Rio de Janeiro.

We also understand that future studies are needed to qualitatively analyze the impact of this type of injury on the police officer’s life and its social consequences.

## Conflicts of interest

The authors declare no conflicts of interest.
